# Estimating the Effects of Obesity and Weight Change on Mortality Using a Dynamic Causal Model

**DOI:** 10.1371/journal.pone.0129946

**Published:** 2015-06-25

**Authors:** Bochen Cao

**Affiliations:** Population Studies Center, University of Pennsylvania, Philadelphia, Pennsylvania, United States of America; Heart Research Institute, AUSTRALIA

## Abstract

**Background:**

A well-known challenge in estimating the mortality risks of obesity is reverse causality attributable to illness-associated and smoking-associated weight loss. Given that the likelihood of chronic and acute illnesses rises with age, reverse causality is most threatening to estimates derived from elderly populations.

**Methods:**

I analyzed data from 12,523 respondents over 50 years old from a nationally representative longitudinal dataset, the Health and Retirement Study (HRS). The effects of both baseline body weight and time-varying weight change on mortality are estimated, adjusting for demographic and socio-economic variables, as well as time-varying confounders including illness and smoking. Body weight is measured by body mass index (BMI). In survival models for mortality, illness and smoking were lagged to minimize bias from reverse causality in estimates of the effect of weight change. Furthermore, because illness both causes and is caused by changes in BMI, I used a marginal structural model (MSM) rather than standard adjustment to control confounding by this and other time-dependent factors.

**Results:**

Overall, relative to normal weight, underweight and Class II/III at baseline are associated with hazard ratios that are 2.07 (95% confidence interval (CI): 1.28–3.37) and 1.82 (1.54–2.16) respectively, whereas overweight and Class I obesity do not significantly lower or raise the mortality risks. Furthermore, relative to stable weight change, all types of weight change lead to significantly increased risk of mortality. Specifically, large weight loss results in a mortality risk that is nearly 3.86 (3.26–4.58) times of staying in the stable weight range and small weight loss is about 1.81 (1.55–2.11 ) times riskier. In contrast, large weight gain and small weight gain are associated with hazard ratios that are 1.98 (1.67–2.35) and 1.20 (1.02–1.41) respectively.

**Conclusions:**

Being underweight or severe obese at baseline is associated with excess mortality risk, and weight change tend to raise mortality risk. Both the confounding by illness and by smoking lead to overestimates of the effects of being underweight at baseline and of weight loss, but underestimates the effect of being obese at baseline.

## Introduction

Many studies investigate the association between obesity and mortality[1–9]. Although it is commonly agreed that low body weight and severe obesity are associated with increased mortality risks, whether overweight and moderate obesity is protective or hazardous are still in debate[7,9–11]. A well-known challenge in estimating the mortality risks of obesity is reverse causality attributable to illness-associated weight loss. Similarly, many smoking-related illnesses (chronic obstructive pulmonary disease (COPD), many cancers and renal disease) are associated with disease-induced weight loss and estimates among smokers are also thought to be highly influenced by reverse causal processes [12–16].

To date, most attempts to deal with reverse causality are inadequate[17]. The most common practices to account for reverse causality in longitudinal studies are excluding subjects with preexisting diseases at baseline[18–20], excluding subjects that experienced substantial weight loss (> = 10lb) in previous years[21,22], and excluding the first few years of follow-up in order to eliminate premature deaths that were potentially caused by illness present at baseline[20,22,23]. These strategies often lead to waste of substantial amount of data, and deletion of large proportion of deaths, reducing statistical power. Additionally, most studies only use a single measurement for weight status, confounders, and other relevant covariates measured at baseline. Some studies show that instead of using weight status taken at only one point in time, change in body weight is a stronger predictor of mortality risks, especially for the elderly[24–26].

Diseases that cause weight loss are best thought of as time-varying confounders to the body weight and mortality association because they fulfill three criteria: (1) body weight predicts the onset of disease, (2) disease predicts subsequent body weight; and (3) diseases are themselves independently predictive of mortality. Thus, diseases operate as both confounders and mediators along the causal pathway, making conventional analytical methods produce biased estimates[27,28]. Smoking also confounds weight change, but in contrast to illness is probably not affected by weight change and thus does not mediate its effects. The complexity of the causal pathway can be seen in Figure A in S1 File.

The present study attempts to address the time-varying confounding of the body weight and mortality association by applying a marginal structural model (MSM)[27–29] to a nationally representative sample. I specifically fill the gaps in existing literature by: (1) modeling both baseline weight and time-varying weight change using multiple waves of interviews, and (2) treating both incident illness and health behaviors as time-varying confounders. To my knowledge, no prior study has used a marginal structural model to estimate the mortality risks of obesity and body weight change.

## Materials and Methods

### Sample

Data for this study are extracted from a nationally representative dataset, the Health and Retirement Study (HRS). The HRS is sponsored by the National Institute on Aging (grant number NIA U01AG009740) and is conducted by the University of Michigan. It is a longitudinal survey of Americans aged 50 and above[30]. Analyses in this study are performed with the RAND HRS data, version L[31]. As a prospective study, HRS serves the purpose of this study better than retrospective studies that are used in many prior studies. Each respondent’s current body weight, health conditions, health behaviors, as well as other relevant characteristics are asked at each HRS interview. In contrast, retrospective studies rely on respondents’ recalling their personal history, thus making it less possible to collect data that is as accurate and contains as many repeated measures for relevant variables as the HRS does. Additionally, retrospective studies are more likely to have selection bias problem. For instance, the individuals who were severely obese may not have survived to the survey to report their weight history.

In this paper, I only include the initial HRS cohort that born in 1931–1941 and entered survey in 1992, and the War Babies (WB) cohort that born in 1942–1947 and entered survey in 1998. These two cohorts entered the survey at approximately same age (50–60 years old) and are both interviewed every two years.

The initial HRS cohort and the WB cohort have a sample size of 9,763 and 2,760 respectively, making a total of 12,523 respondents. In order to calculate weight change between subsequent interviews, I exclude those who died (N = 180) or dropped out of the study (N = 1,551) before the second interview, and those whose body mass index (BMI) is missing (N = 1,172) in any interview. I further exclude outliers that with very high baseline BMI (>60) (N = 3) and that have experienced extraordinary weight loss (>30% of body weight) (N = 939) between two consecutive interviews. These exclusions combined lead to a sample of 8,678 respondents and 67,772 observations (person-interviews).

### Weight Variables

Body weight is measured in BMI (kg/m^2^), which is calculated from self-reported weight and height at each interview. Five levels of body weight are categorized according to the guidelines provided by World Health Organization[32], including Underweight (BMI<18.5), Normal Weight (18.5< = BMI<25), Overweight (25< = BMI<30), Class I Obese (30< = BMI<35) and Class II/III Obese (BMI> = 35). Weight change is measured by the percentage change in BMI between any two consecutive interviews and is classified into five categories: Large Weight Loss (BMI drops by > = 10%), Small Weight Loss (BMI drops by [5%, 10%]), Stable Weight Change (BMI changes by (-5%, 5%)), Small Weight Gain (BMI increases by [5%, 10%)), and Large Weight Gain (BMI increases by > = 10%). In this analysis, Normal Weight and Stable Weight Change are used as reference groups.

### Confounders

A dummy variable for preexisting diseases is constructed using self-reported diagnosis of chronic diseases at each interview. If a respondent answers yes at an interview to any of the questions that ask whether he/she has been told that he/she has one of the five types of chronic disease (diabetes, cancers, lung diseases, heart problems and stroke) ever (for the first interview) or since last interview (for the subsequent interviews), he/she will be marked as having had preexisting diseases. In addition, self-rated health is included to account for unobservable factors that can not be easily measured or diagnosed. The original variable in HRS has five categories (excellent, very good, good, fair and poor) and is re-grouped into two. The first three categories (excellent, very good and good) are combined to indicate good self-rated health, while the other two (fair and poor) are combined to indicate poor self-rated health and used as the reference group.

Three dummy variables are created based on individuals’ smoking status, whether one has never smoked or previously smoked or is currently smoking, at each interview. Never smoker is used as the reference group.

In addition, a dummy variable for physical activity and exercise is created based on the frequency of vigorous physical activity. However, since this frequency is measured differently in the first six and the rest four rounds of interviews, the dummy variable is assigned one if the respondent had vigorous physical activity three or more times a week in the first six interviews, or two or more times a week in the other interviews.

Some time-independent characteristics are adjusted in this analysis as well. Those include gender, age at first interview, race/ethnicity (non-Hispanic white, non-Hispanic black, Hispanic and Other), education levels (no high school diploma, high school diploma or GED, some college and college degree).

Furthermore, several time-dependent socio-economic (SES) covariates that are measured at both first and subsequent interviews, including marital status (never married, married, divorced or separated, and widowed), and log of household income/wealth are also controlled. All time-dependent covariates that are measured subsequent to baseline are lagged for one interview, in order to ensure these risk factors and weight change line up in the correct order along the causal pathway. On the other hand, time-dependent covariates that are measured at first interview are used as baseline controls in the analytical models.

### Statistical Analyses

As earlier discussed, it is critical to model the association between obesity and mortality dynamically and at the same time properly adjust for the confounding caused by time-varying health conditions and time-varying health behaviors. Because the direct result of sickness or smoking is weight change rather than having a certain level of body weight, I separate one’s BMI measures into two components, baseline BMI and weight change between any two consecutive interviews, by controlling BMI measured at first interview and modeling time-varying weight change.

In addition, standard regression models yield biased estimates of the overall effects of weight change on mortality when there are time-dependent confounders, in particular illness, that affect both mortality and weight change and that are also potentially affected by prior weight change. Specifically, including illness as a time-dependent covariate would adjust away the indirect effect of weight change mediated by its effects on subsequent illness. Instead, I apply a marginal structural model (MSM) that uses inverse weighting to create a pseudo-population in which the association between weight change and mortality is un-confounded[27,28]. The MSM extends methods using propensity scores as inverse probability weights to longitudinal data with time-dependent exposures and confounders[27]. The basic idea of MSM is to weight the sample from the observational longitudinal study with the inverse probability of an individual experiencing a certain level of weight change between two interviews conditional on the values of confounders and other covariates. This weighting resembles a randomized experiment where time-dependent weight changes are thought to be assigned to an individual randomly and therefore the confounding by health conditions is eliminated. Specifically, a single weight is generated for each observation during this procedure. The newly created weights are then supplied to models that estimate the effects of obesity and weight change on mortality. A detailed discussion of the MSM approach used in this paper is presented in S1 File. All analyses are performed with SAS program Proc Genmod.

## Results

Table 1 presents the baseline characteristics from the whole sample by body weight change history over time during the entire study. Overweight people constitutes the largest group (41.3%) of the sample, followed by people with normal weight (33.6%), while Class II/III obese (7.0%) and underweight (1.1%) are observed in only a small proportion of the sample. A large majority (80.0%) of the sample self-rated their health at baseline as at least good, and 70.8% has never been diagnosed with any of the five types of chronic disease. However, only 35.9% of the sample was free from these chronic diseases through the follow-up years.

**Table 1:** Baseline Characteristics by Weight Change Status through All Interviews.

	Whole Sample (n = 8,678)	Large Weight Loss At Least Once (n = 2,337)	Small Weight Loss At Least Once (n = 4,314)	Stable Weight All Time (n = 1,414)	Small Weight Gain At Least Once (n = 5,062)	Large Weight Gain At Least Once (n = 2,610)
Women (%)	51.0	59.2	53.8	41.0	53.4	60.9
Mean age at first interview, Years	55.1 (3.2)	55.3(3.2)	55.2(3.2)	55.3(3.2)	55.0(3.2)	54.9(3.1)
Death (%)	20.3	27.0	18.6	22.9	15.7	18.7
Follow-up years						
For those who died	10.6(4.7)	11.9(4.4)	12.2(4.2)	7.3(4.1)	12.4(4.2)	12.4(4.2)
For those who were censored	14.8(4.9)	16.1(3.8)	16.1(3.7)	11.2(6.4)	15.9(3.8)	16.0(3.7)
Race/Ethnicity (%)						
White, non-Hispanic	75.4	71.2	73.7	79.0	75.9	72.3
Black, non-Hispanic	15.1	18.4	16.3	12.7	14.5	16.9
Hispanic	7.4	8.7	8.1	6.0	7.6	9.0
Other	2.1	1.8	1.9	2.3	2.1	1.9
Education (%)						
Less than a high school diploma	22.3	28.1	23.8	18.0	22.0	25.8
High school diploma/GED	37.5	37.4	37.3	36.6	37.3	39.0
Some college	20.2	18.5	20.6	19.1	20.8	19.9
College degree or higher	20.0	16.0	18.3	26.3	20.0	15.3
Marital Status (%)						
Married	74.9	71.4	73.8	78.6	75.2	71.3
Never married	4.0	4.0	4.1	3.6	3.9	4.2
Divorced/separated	15.1	17.4	15.4	13.3	14.9	17.4
Widowed	6.0	7.2	6.7	4.5	6.0	7.1
Mean Household Income, $1,000s	54.1(62.1)	44.0(48.3)	51.7(79.5)	64.9(80.6)	53.7(92.3)	49.3(90.6)
Smoking Status (%)						
Never smoker	36.8	34.8	37.0	36.9	37.7	35.6
Former smoker	36.7	34.0	36.0	39.5	36.8	34.0
Current smoker	26.5	31.2	27.1	23.7	25.5	30.5
Vigorous Physical Activity (%, ≥3 times per week)	25.6	22.0	24.4	29.2	25.0	24.8
Baseline BMI Categories (%)						
Underweight (<18.5)	1.1	0.7	1.0	1.5	1.0	1.4
Normal (18.5–24.9)	33.6	25.0	29.3	44.3	32.0	28.4
Overweight (25.0–29.9) 41.3 38.7 43.2 38.5 42.7 40.1	41.3	38.7	43.2	38.5	42.7	40.1
Class I obese (30–34.9) 17.0 23.1 18.9 11.7 17.4 20.7	17.0	23.1	18.9	11.7	17.4	20.7
Class II/III obese (≥35.0) 17.0 23.1 18.9 11.7 17.4 20.7Class II/III obese (≥35.0)	7.0	12.5	7.6	4.0	7.0	9.4
Self-report of health (%)						
Excellent	23.0	16.3	22.3	27.1	23.6	19.1
Very Good	29.4	26.3	28.4	31.6	29.5	27.2
Good	27.2	29.4	28.2	24.5	28.0	29.3
Fair	13.3	17.6	14.3	10.3	13.1	16.1
Poor	7.0	10.4	6.8	6.5	5.8	8.3
Chronic Diseases diagnosed before entering the study (%)						
Diabetes	10.5	14.1	10.7	9.6	8.8	11.0
Cancer	5.2	6.5	5.5	4.5	5.2	5.9
Lung Disease	7.1	9.1	7.2	5.7	6.7	8.3
Heart Problem	12.2	14.0	12.1	11.6	11.8	12.2
Stroke	2.6	3.6	2.4	2.1	2.2	3.0
No preexisting diseases	70.8	65.0	70.1	74.1	72.4	68.5
Chronic Diseases diagnosed during the study (%)						
Diabetes	26.2	34.3	29.5	17.1	27.0	30.9
Cancer	19.6	23.8	21.4	13.7	19.9	21.0
Lung Disease	15.6	21.0	17.1	10.4	15.7	19.9
Heart Problem	31.6	40.4	35.4	22.1	32.7	36.6
Stroke	10.4	16.4	12.2	5.3	10.9	13.6
No preexisting diseases	35.9	24.5	30.2	50.6	34.2	29.2

Notes:

Numbers are percentages unless otherwise noted. Standard deviations for continuous variables are in parentheses. Individuals can appear in multiple weight-change categories.

Also shown in Table 1 is that relative to those who remained of stable weight throughout the study, every type of weight change is more common among current/former smokers, those with less vigorous physical activity, those with worse self-rated health and those with more incidents of chronic disease both prior and during the study.

Table 2 summarizes the distributions of weight change by the health status at the interview immediately before the weight change. Similar to the results in Table 1, weight changes in both directions, especially for weight loss, are strongly associated with previous incidences of chronic diseases and poor health status.

**Table 2:** Distribution of Weight Change by Previous Health Status.

	Diagnosis of Chronic Diseases		Self-Rated Health Status	
	No	Yes	Good	Poor
Large Weight Loss	3.42	6.43	3.86	7.76
Small Weight Loss	8.61	11.93	9.31	12.68
Stable Weight	70.23	66.25	69.11	58.52
Small Weight Gain	12.82	12.98	12.84	13.05
Large Weight Gain	4.92	6.41	4.87	8.00
Chi-Square	363.8 (p<0.001)		501.55 (p<0.001)	

Notes: Numbers reported are percentages

In order to examine whether the association between obesity and mortality is subject to time dependent confounding, I run three pairs of model to test whether 1) health conditions predict weight change, 2) health conditions predict mortality, 3) previous weight change predicts health conditions. Robust standard errors are used to account for within-subject correlation due to repeated measures. Details are presented in S2 FileThe results indicate that health conditions indeed operate as both time-dependent confounder and time-dependent mediator along the causal pathway.

Table 3 presents the estimated hazard ratios (with 95% confidence intervals) of baseline body weight and time-dependent weight changes between consecutive interviews, using Marginal Structural Models. Model 1 is a null model that includes only baseline weight status and weight change history. U-shaped and J-shaped association with mortality are observed for baseline weight status and time-dependent weight change respectively. Underweight and Class II/III obese at baseline are associated with excess mortality risks relative to normal weight, while the effects for overweight and Class I obese are not statistically significant. Similarly, large weight loss, small weight loss, and large weight gain are all associated with higher mortality relative to stable weight change.

**Table 3:** Adjusted Effects of Baseline BMI and Weight Change Over Time on Mortality (Marginal Structural Models).

Parameter	Model 1	Model 2	Model 3	Model 4
Weight Loss 10%+	4.68 *** (4.06,5.40)	4.67 *** (4.04,5.41)	4.29 *** (3.68,5.16)	3.86 *** (3.26,4.58)
Weight Loss 5–10%	2.07 *** (1.80,2.37)	2.00 *** (1.74,2.30)	1.94 *** (1.67,2.24)	1.81 *** (1.55,2.11)
Weight Gain 5–10%	1.14 (0.98,1.34)	1.08 (0.90,1.38)	1.18 * (1.01,1.39)	1.20 * (1.02,1.41)
Weight Gain 10%+	1.88 *** (1.57,2.26)	1.82 *** (1.50,2.20)	1.75 *** (1.45,2.12)	1.98 *** (1.67,2.35)
Underweight	2.72 *** (1.91,3.86)	2.63 *** (1.73,3.96)	2.38 *** (1.58,3.60)	2.07 *** (1.28,3.37)
Overweight	0.99 (0.88,1.11)	0.87 * (0.78,0.98)	0.94 (0.83,1.06)	0.91 (0.80,1.03)
Obese I	1.02 (0.89,1.17)	0.94 (0.81,1.08)	1.05 (0.90,1.21)	1.14 (0.99,1.32)
Obese II/III	1.49 *** (1.26,1.77)	1.42 *** (1.19,1.69)	1.72 *** (1.43,2.06)	1.82 *** (1.54,2.16)

Notes:

Model 1: Includes only baseline weight status and time-dependent weight change.Model 2: Adds SES and socio-demographic covariates (both baseline and time-varying).Model 3: Adds confounding by time-dependent health behaviors.Model 4: Adds confounding by time-dependent health conditions.

*p < .05.

**p < .01.

***p < .001.

Model 2 adjusts for socio-demographic and SES characteristics, measured at first interview and follow-up interviews. The estimates from this model change only slightly from those in Model 1. Overall, the hazard ratios for weight change and baseline weight status for all categories decline as expected, as the covariates adjusted tend to have negative associations with weight status and weight change.

Further controlling time-dependent health behavior covariates (smoking and physical activity) in Model 3, I show how these covariates confound the estimates of mortality risks. Inclusion of these covariates leads to increase in hazard ratios for Class II/III obese at baseline, but decrease for other baseline weight categories and all weight change categories except for small weight gain. Although the amount of physical activity is a time-dependent confounder in this model as it has impact on future weight status as well as mortality and can be affected by past weight and health status, it only has a negligible effect on the estimated hazard ratios (model un-shown). Majority of the change in the estimated hazard ratios between Model 2 and Model 3 is attributable to smoking. On the one hand, because smoking accounts for disproportionate number of those non-obese and those who have experienced weight loss, failure to control for it will result in underestimate of the mortality risk of obesity and overestimate of the mortality risks of being underweight and weight loss, which is consistent to the results in Model 3. On the other hand, it has been shown that cessation of smoking is likely to lead to weight gain[33–37], hence after including a dummy variable for ever-smokers, the decrease in estimated mortality risk for time-dependent weight gain is not implausible. This result may also be explained by prior studies that find active smoking is a modifiable risk factor of type 2 diabetes which is associated with both large weight gain and increased mortality[34,38–40].

Model 4 additionally accounts for both diagnosed and underlying health conditions by including time-dependent measurements for diagnose history of chronic diseases and self-rated health respectively. Because the subsample who were underweight at baseline and who have experienced weight loss consist disproportionately individuals with relatively poor health status, it is expected that once health conditions are adjusted the estimated hazard ratios for weight loss and underweight at baseline will decline, while effects of obesity and weight gain will increase. The results in Model 4 confirm this expectation.

Overall, the estimates produced in Model 4 are consistent with existing literature. With reference to normal weight, underweight and Class II/III at baseline are associated with hazard ratios that are 2.0 and 1.8 respectively, whereas overweight and Class I obesity do not significantly lower or raise the mortality risks. Furthermore, relative to stable weight change, all types of weight change lead to significantly increased risk of mortality. Specifically, large weight loss results in a mortality risk that is nearly four times of staying in the stable weight range and small weight loss is about 1.8 times riskier. In contrast, large weight gain and small weight gain are associated with hazard ratios that are 2.0 and 1.2 respectively. These large hazard ratios indicate that weight change has stronger effects on mortality than baseline BMI.

One underlying assumption of the results shown in Table 3 is that the effects of baseline weight and weight change do not vary by smoking status. Table 4 presents the results of analyses stratified by this factor. A model that includes the interactions of baseline smoking history and baseline weight status as well as time-dependent weight changes is also tested. The p values of the interactions are shown in the parentheses below. The effect of being underweight on mortality risk is much stronger among ever smokers (p value for interaction 0.006). The effects of weight loss also appear nominally stronger among ever smokers, but the differences are consistent with chance variation (p value for interaction <0.001).

**Table 4:** Adjusted Effects of Baseline BMI and Weight Change Over Time on Mortality (Marginal Structural Models by Smoking Status).

Parameter	Ever-Smokers (N = 5,483)	Never-Smokers (N = 3,195)
Weight Loss 10%+	4.09 *** (3.03,5.47)	3.75 *** (3.05,4.61)
Weight Loss 5–10%	1.98 *** (1.52,2.56)	1.72 *** (1.42,2.08)
Weight Gain 5–10%	1.43 ** (1.11,1.85)	1.08 (0.88,1.33)
Weight Gain 10%+	2.12 *** (1.57,2.89)	1.90 *** (1.55,2.35)
Underweight	4.23 *** (2.02,8.55)	1.85 *** (1.08,3.03)
Overweight	0.96 (0.76,1.21)	0.88 (0.76,1.02)
Obese I	1.09 (0.89,1.34)	1.01 (0.89,1.15)
Obese II/III	1.74 ** (1.29,2.35)	1.89 *** (1.54,2.34)

Notes:

Both models are built on Marginal Structural Model that includes covariates for SES and socio-demographic characteristics (gender, age at first interview, race/ethnicity, education, and household income), covariates

for health behaviors (physical activity), and covariates for health conditions (previous diagnosis of chronic

diseases and self-rated health conditions)

*p < .05.

**p < .01.

***p < .001.

Similarly, the effects of weight change could vary by baseline weight. Table 5 presents the results of analyses stratified by this factor. Large weight loss is associated with excess mortality across all baseline weight categories, notably among the overweight (p value for interaction 0.045) and obese (p value for interaction 0.029), while large weight gain is a statistically significant indicator of increased mortality only in those groups (p values for interaction 0.038 and <0.001 respectively). Small weight loss is also associated with higher mortality risk for people in the normal (p value for interaction 0.018) and overweight range (p value for interaction <0.001) at baseline. Finally, small weight gains were significantly associated mortality risk only among the obese (P for interaction 0.049). Overall, the estimated effects of weight change on mortality in this analysis are consistent with estimates from the whole sample, and revealed little persuasive evidence for modification of the effects of weight change by baseline weight. This is also confirmed by model that includes interactions of baseline weight status and time-dependent weight changes. The two obesity categories are collapsed into one to reduce the complexity of the model. And the p values for the interactions are reported in the parentheses above.

**Table 5:** Marginal Structural Models by Baseline Weight Status.

Parameter	Underweight	Normal	Overweight	Class I Obese	Class II/III Obese
Weight Loss 10%+	2.65 ** (1.40,5.03)	2.31 ** (1.32,4.05)	4.10 *** (3.10,5.40)	4.16*** (3.29,5.25)	4.60 *** (2.59,8.05)
Weight Loss 5–10%	1.03 (0.50,2.10)	1.42* (1.11,2.09)	1.84 *** (1.45,2.34)	1.34 (0.93,1.96)	1.45 (0.87,2.44)
Weight Gain 5–10%	0.52 (0.11,2.45)	1.20 (0.84,1.71)	1.06 (0.81,1.40)	1.77 *** (1.30,2.41)	1.13 *** (0.73,1.74)
Weight Gain 10%+	0.89 (0.15,5.27)	1.47 (0.97,2.22)	1.75 ** (1.23,2.50)	3.02 *** (1.99,4.59)	2.45 ** (1.81,3.90)

Notes:

All models are based on Model 4 in Table 3.

*p < .05.

**p < .01.

***p < .001.

To motivate use of MSMs for this analysis, Models in Table 3 are replicated using Cox hazard model with time-dependent covariates to demonstrate how standard regression models bias the estimates. Essentially, compared to results from the MSMs, those estimated using the Cox model are closer to the null, consistent with our hypothesis that the standard approach adjusts away indirect effects of exposure. Details are presented in S3 File.

### Sensitivity Analyses

I perform sensitivity analyses to test the robustness of the associations between time-dependent weight change as well as baseline weight status and mortality estimated above, by applying alternative sample- and model- specifications. The remaining parts of the models other than the altered parts are kept unchanged.

Overall, the sensitivity analyses suggest the associations observed from the main model is fairly robust, and all results are consistent with the finding that being underweight and class II/III obese at baseline, as well as weight change in all directions are associated with increased mortality risk, relative to the reference groups. Although variations in estimates of hazard ratios are observed in some cases, they are not unacceptably large. And this variation is most likely due to much smaller sample sizes which lead to less precise estimates and loss of statistical power. Details are discussed in S4 File.

## Discussion

In this study, the confounding by health behaviors and health conditions is adjusted in a time-dependent manner along the causal pathway through application of marginal structural models. As expected, the findings demonstrate that both the confounding by health behaviors and by health conditions lead to overestimates of the effects of being underweight at baseline and of weight loss, but underestimates the effect of being obese at baseline. These results confirm the hypothesis that smoking and sickness often induce low weight and weight loss[7,12,41], and as a result bias estimates of the mortality effects of obesity and weight change. In addition, subsample analysis demonstrates that the confounding by poor health conditions has greater impact on smokers than never-smokers, as smoking is associated with excess risks of developing many chronic diseases which may in turn lead to weight loss and higher mortality.

A major strength of this study is addressing the interrelated association between health conditions and weight change in a dynamic framework, while controlling baseline weight status. It is shown that although being underweight or Class II/III obese at baseline is associated with excess mortality risk, being overweight or moderate obese is not. On the other hand, relative to stable weight change, all other levels of weight change significantly raise mortality risk. The association is J-shaped, with large weight loss being nearly twice as riskier as large weight gain. These results are consistent with Mikkelsen et al (1999) who finds both weight level and weight change have independent U-Shaped associations with mortality, adjusting for smoking and excluding individuals with preexisting and subclinical diseases. Also, compared to BMI measured at baseline, weight change over time is indeed a stronger predictor of mortality. The estimates from this analysis are in general also consistent with Myrskylä and Chang (2009) who use quadratic terms of BMI and weight change measured by the number of units of change in BMI from only the first two interviews. But this present study also shows that weight change will be associated with excess mortality risk for more groups of people depending on their baseline weight status and scale of weight change.

When the subsample analysis is performed by initial weight status, the J-shaped association is generally preserved. This seems to be in contrary to the hypothesis that weight loss for obese or overweight individuals is associated with decreased mortality risk, relative to their initial weight status[42]. However, given that the reference group contains weight loss under 5%, these results are not inconsistent with prior studies[24]. In addition, several observational studies have shown that even voluntary weight loss, such as dieting and exercise, may be associated with increased mortality even for those who are overweight[39,43–45]. Also, many practices for losing weight such as diet drug, fasting and smoking are known to have adverse effect. Moreover, failing to maintain weight at a stable level may indicate poor homeostatic control, which may be associated with functional impairment and mortality, and may indirectly make unhealthy people lose even more weight, leading to higher mortality risk[44,45]. In contrast, large weight gain is associated with increased mortality only for those at or above overweight level, and small weight gain increases mortality only for those who are at least Class I obese. As shown in Table 1, there is much larger proportion of underweight or normal weight people losing weight than the proportion of obese people gaining weight, thus more concerns should be paid to weight loss.

The present study has several limitations. First, BMI measures are constructed by self-reported heights and weights, which are found to often underestimate BMI in prior studies[46], although strong correlation between self-reported and clinically measured BMI have been found in many studies[24,47]. Moreover, BMI may not be an accurate measure for obesity, as its incapacity in directly measuring muscle composition and body fat. Other measurements of fitness such as waist circumference and percent body fat, although not available in HRS data, may better reflect body fatness and hence may be prognostic[48,49]. Second, heterogeneity in mortality related to weight change may exist across different diseases. Despite the fact that HRS provides diagnosis history for many diseases, without information on cause of death it is unreasonable to infer deaths are caused by a certain type of diagnosed disease. Moreover, because of availability of data and the size of sample, the disease variables cannot be more sophisticatedly constructed. This may lead to inadequately adjusted confounding of weight change associated with preexisting chronic diseases. Third, the HRS does not provide measures for historical body weight, such as BMI at age 25 or maximum weight observed; therefore it is impossible to investigate the mortality risk of younger age obesity. Fourth, although the marginal structural model can minimize biases introduced by time-dependent observable confounders, it still demands appropriate model specifications and, as other statistical approaches, it cannot deal with unobservable confounders.

In summary, after addressing some of the concerns in the literature by applying a time-dependent causal model, the findings from this present study suggest that BMI at baseline has a U-shaped association with mortality among elderlies. Additionally, adverse effects are shown for weight change larger than 5% of prior body weight. Future research may investigate the association between obesity and cause-specific mortality, adjusting for confounding by specific types of disease, as some diseases (e.g. diabetes) are more prone to be related to obesity.

## Supporting Information

S1 File Supporting Information 1. (DOCX)

S2 File Supporting Information 2. (DOCX)

S3 File Supporting Information 3. (DOCX)

S4 File Supporting Information 4. (DOCX)
