# Supplementary material for: Estimating the Effects of Obesity and Weight Change on Mortality Using a Dynamic Causal Model
Source: PLoS One. 2015 Jun 25;10(6):e0129946. doi: 10.1371/journal.pone.0129946 (PMC4481504; doi:10.1371/journal.pone.0129946)
Supplement: S1 File — (DOCX) [file pone.0129946.s001.docx]

**Supporting Information 1**

In this paper, the association between body weight change and mortality is modeled in a dynamic way. Not only individuals’ body weights tend to vary over time, the association is also likely to be confounded or mediated by both observable and unobservable time-dependent confounders. For instance, the development of chronic diseases is possibly a consequence of prior weight gain and could lead to both subsequent weight loss and mortality. And the confounding caused by chronic diseases could be more complicated when time-dependent health-related behavior, such as smoking, drinking and exercise, are taken in to account. Figure A demonstrates a complex causal pathway, given that ∆*W(t)* denotes the individual’s exposure (change in body weight) between time *t* and *t+1*, *O(t)* and *U(t)* denote all observable (e.g. diagnosed diseases) and unobservable confounders at time *t* respectively, where *O(t)* and *U(t)* temporally precede ∆*W(t)*, and D stands for the final outcome (mortality). As shown in Figure A, one’s body weight change at a certain time point could influences his/her mortality through both direct and indirect causal pathways along the timeline, and this relation is subject to both observable and unobservable confounding from earlier time periods. In addition, one’s current body weight change could be influenced by the past changes.

However, conventional regression approaches that estimate the effects of change in weight on mortality yield biased estimates when there are time-dependent confounders, such as some chronic diseases, which are influenced by previous weight change, even if these confounders are adjusted in regression models. Consequently, adopting an appropriate model that accounts for the variations of body weight over time and adjusts for time-dependent confounding is essential in this study.

Suppose the association between weight change and mortality is not biased by either observable or unobservable confounders, then the effects of weight change on mortality can be considered causal and direct. In other words, not only there is no history of unobservable confounders *U(0), U(1), U(2), ……, U(t-1),* and *U(t)*, at each time point *t*, one’s past weight change history ∆*W(1),* ∆*W(2), ……,* ∆*W* (t) is also not associated with the history of measured confounders *O(1),O(2) ……, O(t).* To simplify the model, let us assume there is only one data point for each individual. The primary goal is to estimate the adjusted effect of experiencing a certain level of weight change (∆*W_i_*) on mortality outcome (*D_i_*) for individual *i* after sufficiently adjusted for all confounders and risk factors (*O_i_*) including health conditions and health behaviors, assuming there is no other unobservable confounder. In this case, the probability for individual falling into different weight change categories (large weight loss, small weight loss, stable weight change, small weight gain, and large weight gain) would be the same, as if an individual is assigned the amount of weight change randomly in an experiment. This being assumed, one can use the risk ratios to measure the casual effect of one’s weight change being at a certain level as opposed to being at another level on mortality. Let *D_i_(*∆*w)* denote the counterfactual mortality outcome of individual *i* if his level of weight change is set to ∆*W_i_=*∆*w*. The risk ratio that compares mortality for this counterfactual outcome to mortality observed at level ∆*W_i_=*∆*w** can be written as $RR(\Delta w,\Delta w^{*})=\frac{P(D_{i}(\Delta w)=1)}{P(D_{i}(\Delta w^{*})=1)}$, where $P(D_{i}(\Delta w)=1)$ an*d* $P(D_{i}(\Delta w^{*})=1)$, are the probabilities that an individual dies given the levels of weight change are ∆*w* and ∆*w^*^* respectively. This risk ratio can be estimated by fitting a log-linear model as below using the observed data

$\log[P(D_{i}=1|\Delta W_{i}=\Delta w_{i})]=\beta_{0}+\beta_{1}\Delta W_{i}$

where ∆*w_i_* represents the observed weight change for individual *i*. The parameter *β_1_* gives an unbiased estimate of the causal effect of weight change on mortality, given the assumption so far is that confounders do not exist.

However, since the association between weight change and mortality is in fact confounded, the above model will fail to produce an unbiased estimate and the probability of an individual having a certain level of weight change will be conditional on the confounders. To adjust the bias, a pseudo-population, in which individuals’ weight changes are uniformly distributed with equal probability as if they are randomly assigned, can be created by applying a weight that is the inverse of the probability of one’s weight change being at a certain level conditional on his/her vector of all confounders and other relevant covariates as observed in the data set, again assuming all confounders can be adjusted using information provided by the observational dataset and unobservable confounders do not exist. The weight can be expressed as $wt_{i}=\frac{1}{P(\Delta W_{i}=\Delta w_{i}|O_{i}=o_{i})}$, where ∆*w_i_* and *o_i_* are observed weight change and observed confounders and other relevant covariates for individual *i*. This weight can be estimated from observed data using logistic regression for ∆*W* with binomial values, multinomial logistic regression for ∆*W* with multi-level values, and ordinary linear regression for ∆*W* with continuous values. The corresponding function form of ∆*W* will be on the left-hand side of the equation as the dependent variable and the vector of *O* will be on the right-hand side of the equation as a set of independent variables.

This idea of weighting described above can be extended to a Marginal Structural Model (James M Robins, 1998; Robins JM, 1999) which can be applied to longitudinal dataset. In essential, Marginal Structural Model (MSM) is a weighted longitudinal analysis. It treats the exposure/ treatment, in this case the levels of weight change, as a time-dependent covariate. For this study, the key idea therefore is to conduct survival analyses with a set of weights that have been calculated for each individual at each interview.

A typical Cox proportional hazard model with time-dependent treatment variables but no time-dependent confounders is expressed in the form of:

$$h(t|\Delta\bar{W}(t),V)=h_{0}(t)(\partial_{1}\Delta W(t)+\partial_{2}V)$$

Where $\Delta\bar{W}(t)$ represents the observed history of one’s weight change over time up to time *t* (the overbar notation is also adopted for time-dependent confounders *O(t)*), and *V* represents one’s observed time-independent characteristics at baseline, such as gender, race and age at baseline, that do not vary over time, and $h_{0}(t)$ is the unspecified underlying hazard. When time-dependent confounders are not absent, particularly when the time-dependent confounders are both influenced by prior weight changes and are predictors for the subsequent weight changes and final mortality outcome, the causal effect of weight change on mortality will be biased (Hernán & Robins, 2006; J M Robins et al., 2000). In order to correctly adjust for the time-dependent confounders and obtain unbiased causal effect estimates, appropriate weights need to be applied in estimating parameters in the hazard function. Parameters estimated using MSM are called inverse-probability-of-treatment-weighted (IPTW) estimators. However, because the conditional probability of having a certain level of weight change$P(\Delta W_{i}=\Delta w_{i}|O_{i}=o_{i})$ may have large variations when time-dependent confounders *O_i_* are strongly associated with ∆*W_i_*, large weights may be produced for some observations and make the weighted analysis dominated by these observations. Therefore, instead of using the inverse of the conditional probability of the treatment, Robins et al. (2000) proposes to use “stabilized” weights that can reduce the variations of the IPTW estimators by replacing the numerator, 1, with the conditional probability of having a certain level of weight change conditional on weight change history and time-independent characteristics. For subject *i*, the stabilized weights at time *t* is

$$sw_{i}(t)=\prod_{k=0}^{t} \frac{P(\Delta W_{i}(k)=\Delta w_{i}(k)|\Delta\bar{W}_{i}(k-1)=\Delta\bar{w}_{i}(k-1),V_{i}=v_{i})}{P(\Delta W_{i}(k)=\Delta w_{i}(k)|\Delta\bar{W}_{i}(k-1)=\Delta\bar{w}_{i}(k-1),\bar{O}_{i}(k)=\bar{o}_{i}(k))}$$

where *k* is an integer that denotes the number of corresponding time unit (day, month, year, etc). The maximum value of *k* is the integer less or equal to time *t*. $\Delta\bar{W}_{i}(k-1)=(\Delta W_{i}(0),\Delta W_{i}(1),...,\Delta W_{i}(k-1))$*,*represents the subject’s entire weight change history up to the *(k-1)*th time unit, and $\bar{O}_{i}(k)=(O_{i}(1),O_{i}(2),...,O_{i}(k))$*,* denotes subject *i*’s entire history of time-dependent observable risk factors including time-dependent confounders up to the *k*th time unit. $V_{i}$denotes one’s time-independent characteristics at baseline and is contained in $O_{i}(1)$. In the present study, *O_i_* includes age, time-varying smoking status, time-varying health status (diagnosed/ self-rated), time-varying physical activity, time-varying income, and time-varying marital status. While *V_i_* includes race, gender, education attainment, as well as the baseline values for the time-dependent covariates,

One special feature for longitudinal observational datasets is that not every subject’s final outcome is observed. Censoring by loss to follow-up often occurs when the subject drops from the study or when the end of the study has been reached. In order to create a pseudo-population that takes into account not only confounding but also loss to follow-up to make the estimates of the causal effect unbiased, censoring has to be adjusted by introducing an additional set of weights. Let $C_{i}(k)$=1 indicates that the subject *i* is lost to follow-up at the *k*th time unit and $C_{i}(k)$=0 otherwise, the weights can be then expressed as:

$$sw_{i}^{C}(t)=\prod_{k=0}^{t} \frac{P(C_{i}(k)=0|\bar{C}_{i}(k-1)=0,\Delta\bar{W}_{i}(k-1)=\Delta w_{i}(k-1),V_{i}=v_{i})}{P(C_{i}(k)=0|\bar{C}_{i}(k-1)=0,\Delta\bar{W}_{i}(k-1)=\Delta w_{i}(k-1),\bar{O}_{i}(k)=\bar{o}_{i}(k))}$$

Finally, the two weights are multiplied together to get the final weights which will then be used in the models that estimate the effects. Hence, for subject *i,* the weight at time *t* is $wgt_{i}(t)=sw_{i}(t)*sw_{i}^{C}(t)$. Each of the two multiplier weights can be estimated using pooled logistic regression(J M Robins et al., 2000) that treats each person-time unit as an individual observation.

The last step is to estimate the effect of weight change on mortality using a hazard model that incorporates the above weight. However, most statistical packages for estimating Cox proportional model do not support weights that vary over time. One way to circumvent this is to apply weighted pooled logistic regression which has been proved to be equivalent to Cox proportional hazard model(D’Agostino et al., 1990), controlling for baseline hazard and baseline covariates. Let $D_{i}(k)=1$ represents that subject *i* died by the *k*th time unit, and $D_{i}(k)=0$ otherwise, the pooled logistic regression can be written as:

*Logit* $P[D_{i}(k)=1|D_{i}(k-1)=0,\bar{A}_{i}(k-1)=\bar{a}_{i}(k-1),V_{i}=v_{i}]=\lambda_{0}(k)+\lambda_{1}*A_{i}(k-1)+\lambda_{2}V_{i}$

where $\lambda_{0}(k)$ is the unspecified baseline hazard at the *k*th time unit, $\lambda_{1}$ is the coefficient vector for the degrees of weight change, and $\lambda_{2}$is the coefficient vector for the baseline covariates. The hazard ratio can be obtained by simply taking the exponent of corresponding coefficients. Time-dependent confounders have already contributed in calculating the stabilized weights and therefore are not included in the final model, in order to avoid the over-adjustment problem. The above models for estimating weights and the effects of weight change on mortality can be fitted with SAS Proc Genmod.

**References**

1 Robins JM. Marginal structural models. *1997 Proc Sect Bayesian Stat Sci Alexandria, VA Am Stat Assoc* 1998; : 1–10.

2 Robins JM. Marginal structural models versus structural nested models as tools for causal inference. *Halloran E, Berry D, eds Stat Model Epidemiol Environ Clin Trials New York Springer- Verlag* 1999; : 95–134.

3 Hernán M a, Robins JM. Estimating causal effects from epidemiological data. *J Epidemiol Community Health* 2006; **60**: 578–86.

4 Robins JM, Hernan MA, Brumback B. Marginal structural models and causal inference in epidemiology. *Epidemiology* 2000; **11**: 550–560.

5 D’Agostino RB, Lee ML, Belanger AJ, Cupples LA, Anderson K, Kannel WB. Relation of pooled logistic regression to time dependent Cox regression analysis: the Framingham Heart Study. *Stat Med* 1990; **9**: 1317–1326.

**Figure A: Causal Associations between Weight Change and Mortality**

Observable Confounders (t‐1) O(t‐1)

Observable Confounders (t) O(t)

Weight Change in

(t-1, t )

∆W(t‐1)

Weight Change in

(t, t+1 )

∆W(t)

Death

D

Unobservable Confounders (t‐1) U(t‐1)

Unobservable Confounders (t) U(t)
