# Supplementary material for: Estimating the Effects of Obesity and Weight Change on Mortality Using a Dynamic Causal Model
Source: PLoS One. 2015 Jun 25;10(6):e0129946. doi: 10.1371/journal.pone.0129946 (PMC4481504; doi:10.1371/journal.pone.0129946)
Supplement: S2 File — (DOCX) [file pone.0129946.s002.docx]

**Supporting Information 2**

In order to examine whether pre-existing health conditions are predictors of weight change and mortality, and in the meantime can be predicted by previous weight change so that the time-dependent confounding conditions underlying MSM are satisfied, three pairs of model are estimated. The first pair of models test the hypothesis that weight change can be predicted by pre-existing health conditions, by fitting two multinomial logistic models with categories of weight change between survey *n* (*n>=*2) and *n+*1 as dependent variables, both controlling time-varying smoking status as well as demographic and socio-economic covariates, but one (Model A1) includes dummy variable for diagnosis of chronic diseases between *n-*1 and *n* as an predictor while the other one (Model A0) simply works as a null model. The likelihood ratio test (LR=13.08, p<0.01) of these two models suggests that Model A1 predicts weight change better than the null model. In addition, Model A1 shows that having pre-existing chronic diseases is significantly associated with weight loss (relative risk=1.93, p<0.001).

The second pair of models test whether pre-existing health conditions predict mortality, using Cox hazard models with time-varying covariates that predict the mortality risk. Both models control for baseline weight categories, weight change history, smoking status, as well as demographic and socio-economic covariates, but one is a null model (Model B0) and the other one (Model B1) includes time-varying dummy variable for diagnosis of chronic diseases. The likelihood ratio test of these two models produces a LR=218.85 (p<0.001), and the estimate of the pre-existing chronic disease variable in Model B1 shows an association with higher risk of mortality (hazard ratio=2.43, p<0.001) that is statistically significant, indicating pre-existing chronic diseases do predict mortality.

The third pair of models examine whether weight change predicts incidence of chronic diseases. Two logistic models both controlling baseline weight categories, smoking status, as well as demographic and socio-economic covariates, are fitted. Again, one of the two models (Model C1) includes weight change history, while the other one is the null model (Model C0). The likelihood ratio test (LR=157.91, p<0.001) suggests that prior weight change does predict incidence of chronic diseases. In addition, weight gains (both small size (odds ratio=1.08, p<0.01) and large size (odds ratio=1.26, p<0.001)) are associated with excess risk of experiencing chronic disease.

Alternatively, substituting self-reported health conditions for diagnosis of chronic diseases in all the above models yields similar results, indicating that health conditions (both observable and non-observable) indeed operate as both confounders and mediators along the causal pathway.
