# Supplementary material for: Estimating the Effects of Obesity and Weight Change on Mortality Using a Dynamic Causal Model
Source: PLoS One. 2015 Jun 25;10(6):e0129946. doi: 10.1371/journal.pone.0129946 (PMC4481504; doi:10.1371/journal.pone.0129946)
Supplement: S3 File — (DOCX) [file pone.0129946.s003.docx]

**Supporting Information 3**

In order to demonstrate how standard regression models bias the estimates, I repeated the analyses presented in Table 3, using Cox hazard model with time-dependent covariates. Results are presented in S3 Table.

Comparing to its MSM counterpart, Model 1 estimated using Cox model has a considerably smaller hazard ratio for large weight loss. This is very likely because the MSMs take into account the dependency that between weight changes during consecutive survey windows and that between weight changes and baseline weight status, while the Cox models do not. To test this hypothesis, I regress weight change between survey *n* (*n*>=2) and survey *n*+1 on baseline weight status and weight change between survey *n*-1 and survey *n*. The estimates suggest prior weight gain and being at least Class I obese at baseline are associated with higher chances of weight loss at a later time. In contrast, prior weight loss is associated with future weight gain. As a result, failing to account for these dependencies incline to lead to underestimates of the mortality risks of both weight change and baseline weight status, especially for the extreme levels.

Similar to the MSM, the estimates from Model 2 using Cox model change only slightly from those in model 1. Overall, the hazard ratios for weight change and baseline weight status for all categories decline as expected, as the covariates adjusted tend to have negative associations with weight status and weight change. Adding health behaviors in the Cox model yields similar changes in the estimated hazard ratios at it does for the MSM. Comparing to Model 2, Model 3 shows increase in hazard ratios for Class II/III obese at baseline, but decrease for other baseline weight categories and all weight change categories.

Model 4 additionally adjusts for confounding by health conditions. It is expected that the estimated hazard ratios for weight loss and being underweight at baseline will decline, while effects of obesity and weight gain will increase. In fact, although the effects of weight loss and underweight at baseline on mortality decline as expected, the effects of weight gain and Class II/III obesity at baseline on mortality drop by 4% and over 25% respectively. This is because the Cox hazard model fails to produce unbiased estimates when the time-dependent confounder in this model, health conditions, is simultaneously 1) predicted by past weight or weight change history, 2) a predictor of future weight change and mortality. Simply including time-dependent health status in the Cox model will lead to over-adjustment for the effects of baseline weight status, as time-dependent health status after baseline operates as a mediator between baseline weight status and mortality. This explains why the effect of being underweight at baseline drops by 25.5% from Model 3 to Model 4. Similarly, the over-adjustment could be the reason of the slight decline in the effects of large weight gain as well. In contrast, the MSM includes the time-dependent health condition covariates only in the re-weighting models that calculate the probability a respondent experiencing a certain level of weight change or being censored at a certain time-point, but not in the model to which the new weights are supplied to calculate the direct effects of weight change and baseline weight status on mortality. Consequently, the MSM is free from the over-adjustment problem and produce unbiased estimates for the direct causal effects of weight change and baseline weight status on mortality. In sum, the comparison between Model 4 using MSM and Cox model demonstrates that MSM is the appropriate model to apply when health status operates as a time-dependent confounder. And, indeed, the estimates produced in Model 4 using MSM are consistent with existing literature.

**S3 Table: Adjusted Effects of Baseline BMI and Weight Change Over Time on Mortality
Cox Hazard Models with Time-Varying Covariates**

| **Parameter** | **Model 1** | **Model 2** | **Model 3** | **Model 4** |
| --- | --- | --- | --- | --- |
| Weight Loss 10%+ | 3.95 *** (3.35,4.65) | 3.84 *** (3.26,4.53) | 3.52 *** (2.99,4.16) | 3.10 *** (2.63,3.66) |
| Weight Loss  5-10% | 2.01 *** (1.72,2.35) | 1.95 *** (1.67,2.28) | 1.85 *** (1.58,2.16) | 1.72 *** (1.47,2.02) |
| Weight Gain  5-10% | 1.21 * (1.02,1.44) | 1.22 * (1.02,1.45) | 1.18  (0.99,1.41) | 1.151  (0.97,1.37) |
| Weight Gain 10%+ | 1.83 *** (1.49,2.25) | 1.80 *** (1.46,2.21) | 1.63 *** (1.32,2.01) | 1.55 *** (1.28,1.89) |
| Underweight | 2.41 *** (1.57,3.71) | 2.32 *** (1.50,3.59) | 1.83 ** (1.17,2.85) | 1.75 * (1.12,2.73) |
| Overweight | 1.03 (0.90,1.18) | 0.92 (0.81,1.05) | 0.96 (0.84,1.1) | 0.92 (0.80,1.05) |
| Obese I | 1.14 (0.97,1.34) | 1.02 (0.87,1.20) | 1.11 (0.94,1.31) | 0.97 (0.83,1.15) |
| Obese II/III | 1.38 *** (1.08,1.73) | 1.30 *** (1.03,1.62) | 1.69 *** (1.38,2.07) | 1.26 * (1.03,1.54) |

**Notes:**

Model 1: Includes only baseline weight status and time-dependent weight change.
Model 2: Adds SES and socio-demographic covariates (both baseline and time-varying).
Model 3: Adds confounding by time-dependent health behaviors.
Model 4: Adds confounding by time-dependent health conditions.

**p* < .05. ***p* < .01. ****p* < .001.
