# Supplementary material for: Estimating the Effects of Obesity and Weight Change on Mortality Using a Dynamic Causal Model
Source: PLoS One. 2015 Jun 25;10(6):e0129946. doi: 10.1371/journal.pone.0129946 (PMC4481504; doi:10.1371/journal.pone.0129946)
Supplement: S4 File — (DOCX) [file pone.0129946.s004.docx]

**Supporting Information 4**

I perform sensitivity analyses to test the robustness of the associations between time-dependent weight change as well as baseline weight status and mortality estimated above, by applying alternative sample- and model- specifications. The rest of the models other than the altered parts are kept unchanged. The results are displayed in S4 Table, and are compared with Model 4 in Table 3, the main model.

Model SA1 restricts the sample to subjects that were free from any of the five types of chronic diseases in all interviews they have participated. This restriction ends up with keeping only 3,117 subjects, 21,319 observations and 319 deaths from the original sample. The estimates of hazard ratios for weight gain and being overweight or above at baseline remain relatively constant from the main model, while the estimated effects of weight loss and being underweight at baseline decline but still are associated with increased mortality. Some hazard ratios lose significance in this analysis.

Model SA2 restricts the sample to subjects that have rated their health status as at least good in all surveys they participated. This restriction strategy aims to obtain a subsample that is subject to minimum unobservable confounders and it produces a total of 4,219 subjects (30,168 observations) among which only 377 were observed death. Both small and large weight loss, as well as large weight gain remains to be associated with excess mortality. Class II/III obese at baseline is now associated with only slightly lower mortality risk relative to the model with whole sample, while the effects of other three weight categories lose significance but their point estimates do not change much.

The third sensitivity analysis limits the sample to never smokers at baseline that remained non-smokers until death or dropping off the study (identical to column 2 in Table 4). After exclusion of current and former smokers, the number of observations drops to 23,823 and the number of respondents reduces to 3,195, among whom 346 died. The basic associations continue to exist. As smokers are more likely to be leaner and to die, excluding this group would presumably yield larger hazard ratio for obesity but lower hazard ratio for underweight. It is confirmed by the estimates from this model.

Given that people tend to lose weight during the years closer to death due to illness, the fourth sensitivity analysis attempts to find out how the results would change if this bias is minimized by excluding individuals those died within eight years (four follow-up interviews) after their first interview. Since those who lost weight due to severe illness tend to die in a much shorter period (1-3 years), this restriction using eight years will yield more conservative estimates. This leaves 7,073 respondents, 56,892 observations and 1,145 deaths. All changes in point estimates of hazard ratios are in general considerably small, as regard to the main model. Nevertheless, it is worth to notice that the mortality risk associated with Class I obese becomes statistically significant, and its magnitude increases as expected.

The fifth model uses a quadratic measure instead of dummy variables for BMI measures. As the estimates in previous models, the alternative quadratic BMI measures also show a U-Shaped association with mortality. Again, the hazard ratios associated with time-dependent weight changes remain relatively constant in all categories.

Overall, the sensitivity analyses suggest the associations observed from the main model is fairly robust, and all results are consistent with the finding that being underweight and class II/III obese at baseline, as well as weight change in all directions are associated with increased mortality risk, relative to the reference groups. Although variations in estimates of hazard ratios are observed in some cases, they are not unacceptably large. And this variation is most likely due to much smaller sample sizes which lead to less precise estimates and loss of statistical power.

**S4 Table: Sensitivity Analyses**

| **Parameter** | **Main** | **SA1** | **SA2** | **SA3** | **SA4** | **SA5** |
| --- | --- | --- | --- | --- | --- | --- |
| BMI Square |  |  |  |  |  | 1.01 *** (1.00,1.01) |
| BMI |  |  |  |  |  | 0.75 *** (0.71,0.79) |
| Weight Loss 10%+ | 3.86 *** (3.26,4.58) | 3.13 *** (2.10,4.68) | 3.12 *** (2.14,4.56) | 3.75 *** (3.05,4.61) | 4.22 *** (3.45,5.14) | 3.84 *** (3.28,4.48) |
| Weight Loss 5-10% | 1.81 *** (1.55,2.11) | 1.57 **  (1.12,2.19) | 1.70 *** (1.26,2.29) | 1.72 *** (1.42,2.08) | 1.96 *** (1.64,2.34) | 1.80 *** (1.55,2.08) |
| Weight Gain 5-10% | 1.20 * (1.02,1.41) | 1.15 (0.83,1.61) | 1.07 (0.79,1.45) | 1.08 (0.88,1.33) | 1.18  (0.96,1.45) | 1.29 **  (1.09,1.52) |
| Weight Gain 10%+ | 1.98 *** (1.67,2.35) | 1.89 * (1.58,2.26) | 1.09 (0.66,1.80) | 1.90 *** (1.55,2.35) | 1.77 *** (1.40,2.24) | 1.91 *** (1.57,2.33) |
| Underweight | 2.07 *** (1.28,3.37) | 1.84  (0.76,4.47) | 1.88 (0.62,5.67) | 1.85 *** (1.08,3.03) | 1.94 *  (1.05,3.57) |  |
| Overweight | 0.91 (0.80,1.03) | 0.85. (0.66,1.09) | 0.91 (0.72,1.15) | 0.88  (0.76,1.02) | 1.07  (0.92,1.24) |  |
| Obese I | 1.14 (0.99,1.32) | 1.03 (0.73,1.44) | 1.21  (0.90,1.63) | 1.01 (0.89,1.15) | 1.31 **  (1.10,1.57) |  |
| Obese II/III | 1.82 *** (1.54,2.16) | 1.68  (0.96,2.95) | 1.80 ** (1.25,2.74) | 1.89 *** (1.54,2.34) | 1.90 *** (1.52,2.38) |  |

**Notes:**
Main: Refers to Model 4 in Table 3.
SA1: Sample is restricted to subjects that were free from any of the five types of chronic diseases in all interviews.
SA2: Sample is restricted to subjects that have rated their health status as at least good in all interviews.
SA3: Sample is restricted to never smokers at baseline that remained non-smokers in all interviews.
SA4: Excludes individuals those died within eight years (four follow-up interviews) after their first interview.
SA5: Use quadratic BMI measures for baseline weight status.

**p* < .05. ***p* < .01. ****p* < .001.
